# Impact of Plateletpheresis on the Hemoglobin, Hematocrit, and Total Red Blood Cell Count: An Updated Meta-Analysis

**DOI:** 10.7759/cureus.61510

**Published:** 2024-06-01

**Authors:** Chanchal Ashok, Sunil Mahto, Sushma Kumari, Amit Kumar, Deepankar, Vidyapati, Manoj Prasad, Mayank Mahajan, Partha Kumar Chaudhuri

**Affiliations:** 1 Pathology, Rajendra Institute of Medical Sciences, Ranchi, IND; 2 Blood Bank, Rajendra Institute of Medical Sciences, Ranchi, IND; 3 Laboratory Medicine, Rajendra Institute of Medical Sciences, Ranchi, IND; 4 Ophthalmology, Rajendra Institute of Medical Sciences, Ranchi, IND; 5 Medicine, Rajendra Institute of Medical Sciences, Ranchi, IND; 6 Internal Medicine, Rajendra Institute of Medical Sciences, Ranchi, IND; 7 Paediatrics, Rajendra Institute of Medical Sciences, Ranchi, IND

**Keywords:** meta-analysis, systemic review, plateletpheresis, donor safety, hemoglobin, hematocrit

## Abstract

Plateletpheresis has become a pivotal part of transfusion medicine. With the increasing demand for plateletpheresis, donor safety is an area of concern because plateletpheresis alters donor hematological parameters. For a better understanding of plateletpheresis, a systemic review is needed to study more evidence-based aspects of plateletpheresis. Electronic databases PubMed, Google Scholar, and Cochrane Library were used to find articles from January 1, 1980, to May 23, 2024. The random effect model was used to meta-analyze the effect of plateletpheresis on hematocrit, hemoglobin, and red blood cell (RBC) count. The Preferred Reporting Items for Systematic Reviews and Meta-Analyses (PRISMA) guideline was followed. A total of 24 studies were found; the effect of plateletpheresis on hemoglobin, hematocrit, and RBC count was studied in the following respective numbers of donors: 3,374, 3,374, and 690. A decrease of hemoglobin, hematocrit, and RBC count was observed after plateletpheresis having a weighted mean difference (WMD) of 0.50 (95%CI = -0.72 to -0.27), WMD of -1.36 (95%CI = -2.05 to -0.66), and WMD of -0.18 (95%CI = -0.23 to -0.12), respectively. Plateletpheresis shows a decrease in the value of hematological parameters such as hemoglobin, hematocrit, and erythrocyte count due to blood loss in the kits employed in the procedure; cell lysis was also seen because of exposure of erythrocytes to stress or change in osmotic pressure. Thus, strict criteria for donation must be developed for better safety of the donors. Improved automated cell separators for plateletpheresis should be made available in blood banks to ensure good quality hematologic products. Our findings suggest that the duration of the procedure should be decreased.

## Introduction and background

Platelet transfusion is essential to the care and treatment of patients with cancer, hematopoietic transplant recipients, and surgical patients. Aphaeresis machinery, which are automated cell separators available in India, plays a very important role in the treatment of patients in hospitals. Advances in technologies such as automated cell separators have improved the efficiency, safety, and quality of blood products by apheresis [1]. Apheresis is a procedure in which, first, the blood is withdrawn from the donor and separated into one or more components ex vivo [2]. Single donor platelet (SDP) is a method of obtaining platelets from donors using an automated cell separator machine such as apheresis. Newer generation apheresis has better platelet productivity, so now it is an integral part of modern transfusion practice [3]. Platelets produced as a byproduct of SDP are analogous to six to eight random platelet concentrates. SDP has the benefit of producing leukoreduced products, which leads to compatible human leukocyte antigen and matched platelet antigen phenotypes [2]. The apheresis procedure produces a higher-quality product with less donor exposure and platelet increment in a patient, which improves the clinical scenario for the patient because it quantitatively extracts a greater number of platelets from an individual [4].

The effect on donors after the procedure is always a concern. There have been very few adverse reactions noted. The reaction seen is either local or systemic [5]. Common adverse reactions seen were hematoma, swelling, pain, phlebitis, and vasovagal attack. Adverse reactions seen in patients are minimal because of controlled doses and volume collection. There is less refractoriness and alloimmunization in plateletpheresis as compared to the manual method of extracting platelets. The benefit of plateletpheresis is that the time interval between donations is short, contrary to the manual method [6]. Acid citrate dextrose is used in kits of apheresis, which sometimes causes citrate toxicity [2]. For the therapeutic benefit of the patients, the most crucial factor is the quality of the product produced by apheresis, for which the World Health Organization (WHO) has standardized the requirement of apheresis for the platelet component. According to the American Association of Blood Banks, platelet components produced by apheresis should at least contain 3x10^11^/unit platelets in 90% of the sampled units, and here leucocyte count must be < 5 x 10^6^ cells/mL. European guidelines require that the leucocyte count should be < 1x10^6^ cells/mL [2]. Red cell contamination should be < 0.5 mL. Many studies have been published on plateletpheresis to check the efficiency and quality of blood products such as platelet concentrates and their impact on hematological parameters such as hemoglobin, hematocrit, and red blood cells (RBC) [7-9]. A few authors have mentioned a significant decrease in the complete blood count after the apheresis procedure [6,9]. While there are studies that have shown an increase in hemoglobin, hematocrit, and white blood cells (WBC) after SDP, some studies provide data on a significant fall in hematological parameters [7,8].

## Review

Methods

**Figure 1:**
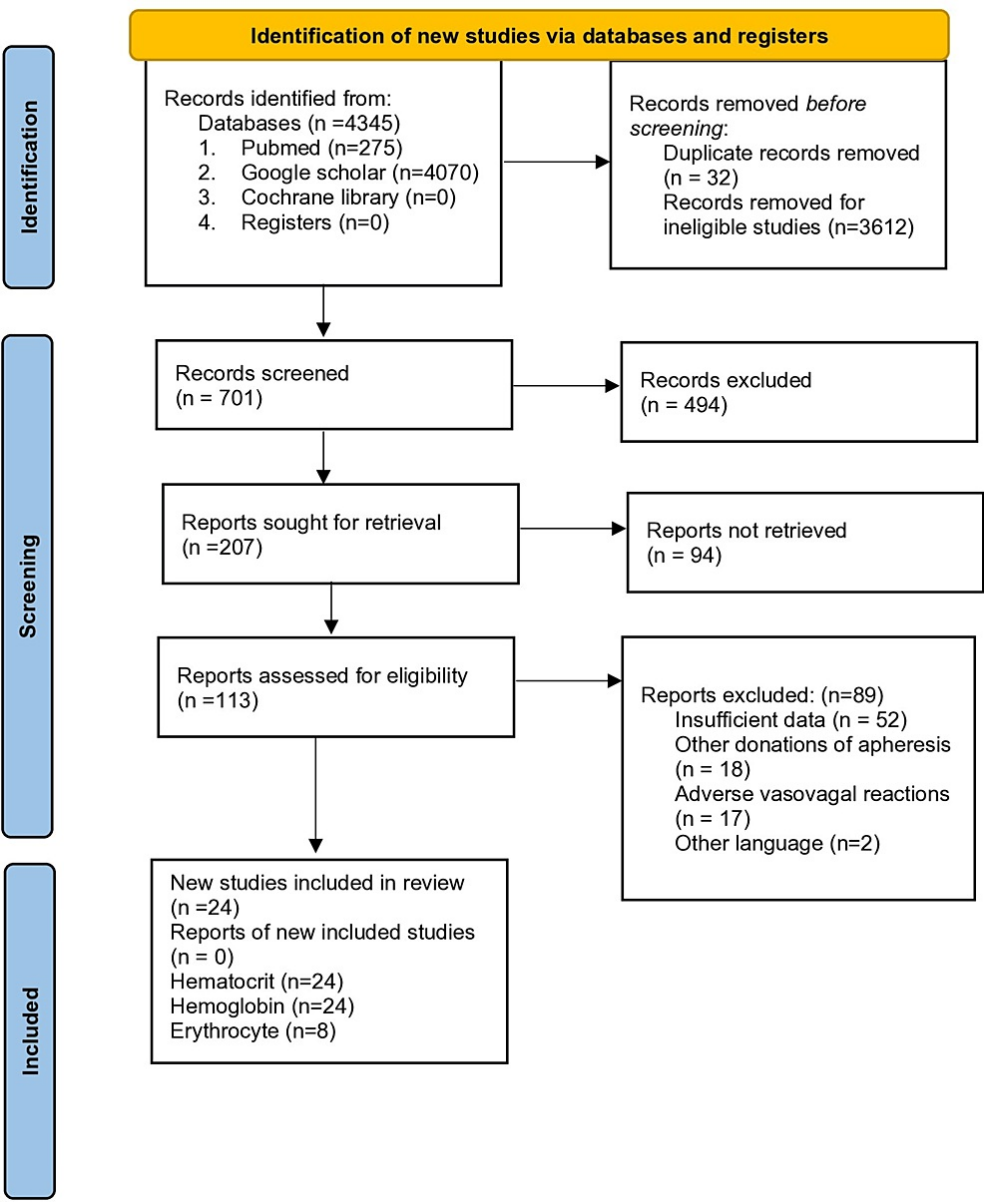
PRISMA flow diagram representing the selection and inclusion of different studies.

This systematic review was registered in the International Prospective Register of Systematic Reviews (PROSPERO 2022, # CRD42022323146) and reported as per the guidelines of the Preferred Reporting Items for Systematic Reviews and Meta-Analysis (PRISMA) (Figure 1).

Type of Study

This is a meta-analysis of a systematic review.

Participants, Intervention, Comparison, Outcome, Study Design (PICO) Criteria

We define the research question by employing the PICO criteria.

Population

The population of this study is plateletpheresis donors.

Intervention

The intervention done in this study is plateletpheresis.

Comparison

The measurements of hemoglobin, hematocrit, and RBC count before and after the procedure of plateletpheresis were compared.

Outcome

We estimated the effect on hematological parameters such as hemoglobin, hematocrit, and RBC count before and after plateletpheresis. Meta-analysis was done on the literature that has shown the effect on hemoglobin, hematocrit, and RBC count, which led to the generation of better-quality evidence on plateletpheresis so that both patients and donor safety measures can be taken by standardizing the criteria of donation.

Inclusion Criteria

Studies included were cross-sectional studies that reported sufficient data about the effect of plateletpheresis on values of hematological parameters such as hematocrit, hemoglobin, and erythrocyte count, reported data in the English language, were conducted in the age group of more than 18 years, included both males and females, and were published as full-text articles and as original articles.

Exclusion Criteria

Studies with inadequate data to compute the pooled effect size, case reports, conference processing, and preprint articles were excluded.

Information Sources

We searched databases such as PubMed, Google Scholar, and the Cochrane Library to obtain the relevant articles or studies published from January 1, 1980, to May 26, 2024. In addition, references from eligible studies were also sought for the relevant articles.

Search Strategy

Keywords used for the search of relevant articles were "Plateletpheresis," "Blood Donation," and "Hematological Parameters". The keywords were combined using the Boolean operator AND. A search filter was applied that restricted results to humans.

Selection Process

Two independent reviewers (CA and AK) searched and selected the published literature and retrieved the desired data from the studies fulfilling the inclusion criteria. Any discrepancy or disagreement was resolved through mutual consensus and discussion.

Risk of Bias and Applicability

A critical appraisal checklist for cross-sectional studies has been done by the Joanna Briggs Institute (JBI). The checklist includes eight questions that inquire about inclusion criteria, subjects and settings, validity and reliability of exposure measurements, standard criteria used for measurement of the condition, confounding factors, strategies to deal with confounding factors, validity and reliability of outcome measurements, and appropriate statistical analysis (Table 1).

**Table 1:** JBI critical appraisal checklist for the cross-sectional studies. Q1. Were the criteria for inclusion in the sample defined? Q2. Were the study subjects and the setting described in detail? Q3. Was the exposure measured in a valid and reliable way? Q4. Were objective, standard criteria used for measurement of the condition? Q5. Were the confounding factors identified? Q6. Were the strategies to deal with confounding factors stated? Q7. Were the outcomes measured in a valid and reliable way? Q8. Was appropriate statistical analysis used? 1=Y, 0=N NA - Not Applicable

Study	Q1^*^	Q2^*^	Q3^*^	Q4^*^	Q5^*^	Q6*	Q7*	Q8^*^
Sahoo et al. [2]	1	1	1	1	NA	NA	1	1
Lewis et al. [7]	1	0	1	1	NA	NA	1	1
Love et al. [8]	1	1	1	1	NA	NA	1	1
Beyan et al. [9]	1	1	1	1	NA	NA	1	1
Katz et al. [10]	1	0	1	1	NA	NA	1	1
Rock et al. [11]	1	1	1	1	NA	NA	1	1
Buchholz et al. [12]	1	1	1	1	NA	NA	1	1
Irfan et al. [13]	1	1	1	1	NA	NA	1	1
Bor-Kucukatay et al. [14]	1	1	1	1	NA	NA	1	1
Das et al. [15]	1	1	1	1	NA	NA	1	1
Moog [16]	1	1	1	1	NA	NA	1	1
Tendulkar et al. [17]	1	1	1	1	NA	NA	1	1
Mahmood et al. [18]	1	1	1	1	NA	NA	1	1
Heuft et al. [19]	1	1	1	1	NA	NA	1	1
Macher et al. [20]	1	1	1	1	NA	NA	1	1
Patidar et al. [21]	1	1	1	1	NA	NA	1	1
Noomani et al. [22]	1	1	1	1	NA	NA	1	1
Sachdeva et al. [23]	1	1	1	1	NA	NA	1	1
Suresh et al. [24]	1	1	1	1	NA	NA	1	1
Gite et al. [25]	1	1	1	1	NA	NA	1	1
Farhat et al. [26]	1	1	1	1	NA	NA	1	1
Landzo et al. [27]	1	1	1	1	NA	NA	1	1
Khurshid et al. [28]	1	0	1	1	NA	NA	1	1
Neha et al. [29]	1	1	1	1	NA	NA	1	1

Statistical Analysis

A standardized mean difference with 95%CI was used to determine pooled effect size. The random effects model was used in case of heterogeneity of more than 50%; otherwise, a fixed effect model was used. P value less than 0.05 was considered statistically significant. Publication bias was determined by the funnel plot and Begg and Egger test. The JBI tool was used for the methodological quality assessment of the cross-sectional studies. The Cochrane nonrandomized quality scale was used for pre- and post-study methodological quality assessment.

Results

**Figure 2:**
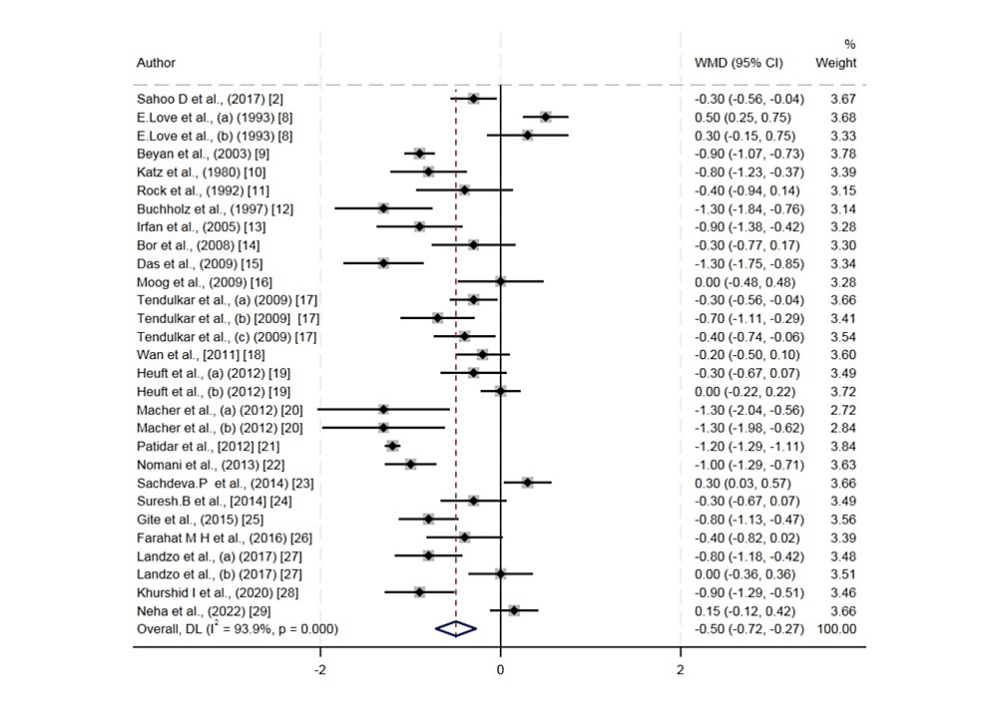
Forest plot for the difference in hemoglobin values between pre-donation and post donation. Refs. [2,8-29]

**Figure 3:**
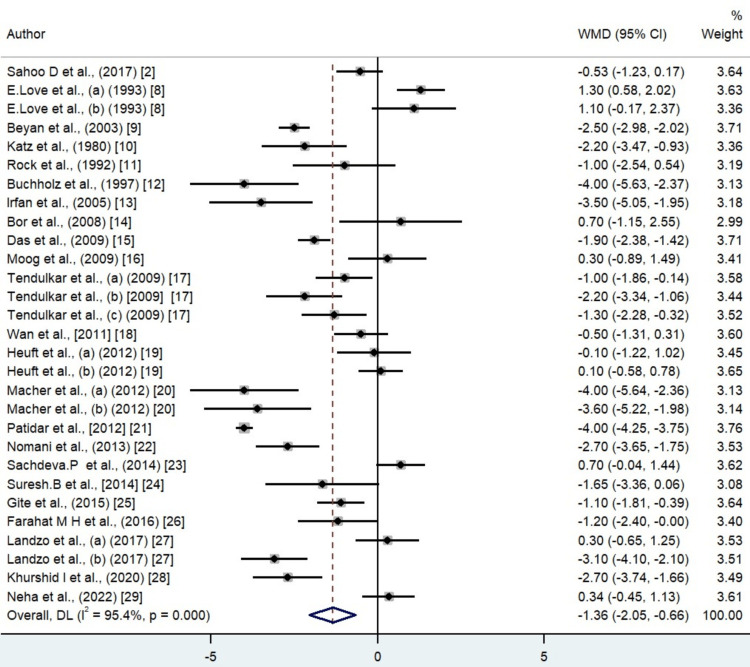
Forest plot for the difference in hematocrit values between pre donation and post donation. Refs. [2,8-29]

**Figure 4:**
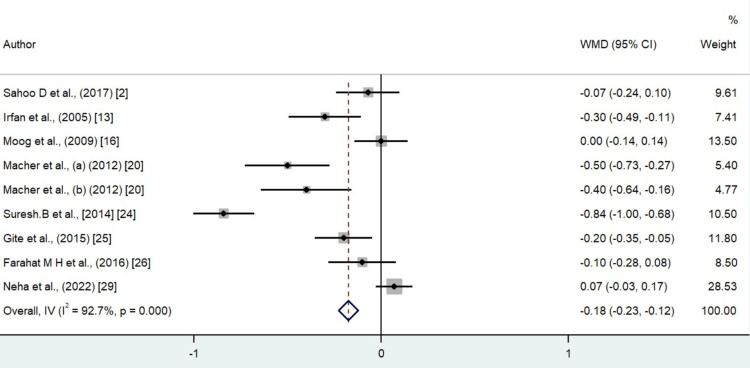
Forest plot for the difference in erythrocyte count values between pre donation and post donation. Refs. [2,13,16,20,24-26,29]

A total of 24 studies were included in the meta-analysis of the systematic review (Table 2). Donors went through a plateletpheresis procedure. The majority of donors were male. The pre- and post-hematological parameters such as hemoglobin, hematocrit, and RBC counts were evaluated. In our study, the analysis showed that the level of hemoglobin decreased up to 0.50 gm/dL after plateletpheresis (95%CI = -0.72 to -0.27). The level of hematocrit also showed a decrease after post donation of platelet by a mean difference of 1.36 (95%CI = -2.05 to -0.66). RBC count also showed a decrease in the level after plateletpheresis by -0.18 × 10^12^/L (95%CI = -0.23 to -0.12). Heterogenicity was found in hemoglobin, hematocrit, and erythrocyte count, but the graph was evenly distributed. The heterogenicity can be confirmed by the value of I^2^ in the random effect model, that is, 93.9% for hemoglobin, 95.4% for hematocrit, and 92.7% for RBCs (Figures 2-4).

**Table 2:** Pre- and post-plateletpheresis donation values of hematocrit, hemoglobin, and erythrocyte count in 24 studies included in the meta-analysis. NA - Data not available in the study (a)*, (b)* - denotes hematological values in the male and female populations, respectively. (i)*, (ii)*, (iii)* - denotes hematological values after plateletpheresis by different cell separators Amicus, Fenwal CS-3000 Plus, and Cobe spectra, respectively. #, ## - denotes hematological values after double plateletpheresis and triple plateletpheresis, respectively. ($), ($$) - denotes hematological values after plateletpheresis by different cell separators Fenwal Amicus and CaridianBCT Trima Accel, respectively. (@), (@@) - denotes hematological values after plateletpheresis by two different methods, i.e., double-needle continuous flow and single-needle intermittent flow, respectively.

Authors	Country	Sample Size	Hematocrit Pre-Donation	Hematocrit Post Donation	Hemoglobin Pre-Donation	Hemoglobin Post Donation	Erythrocytes Pre-Donation	Erythrocytes Post Donation	Ethnicity
Sahoo et al. (2017) [2]	India	135	44.05±2.87	43.52±2.97	14.65±1.09	14.35±1.07	5.44±0.67	5.37±0.75	Indian
Lewis et al. (1991) [7]	UK	92	43.9	43.8	14.3	14.3	NA	NA	Caucasian
Love et al. (a)* (1993) [8]	Britain	78	41.8±2.2	43.1±2.4	14.7±0.7	15.2±0.9	NA	NA	Caucasian
Love et al. (b)* (1993) [8]	Britain	34	38.9±2.4	40±2.9	13.6±0.9	13.9±1.0	NA	NA	Caucasian
Beyan et al. (2003) [9]	Turkey	265	43.7±2.8	41.2±2.8	14.9±1.0	14±1.0	NA	NA	Asian
Katz et al. (1980) [10]	USA	24	42.2±2.2	40±2.3	14.1±0.7	13.3±0.8	NA	NA	Caucasian
Rock et al. (1992) [11]	Canada	13	44±2.0	43±2.0	14.7±0.7	14.3±0.7	NA	NA	Caucasian
Buchholz et al. (1997) [12]	USA	26	40±3.0	36±3.0	13.3±1.0	12±1.0	NA	NA	Caucasian
Irfan et al. (2005) [13]	Turkey	35	46.4±3.0	42.9±3.6	15.1±0.8	14.2±1.2	5.2±0.3	4.9±0.5	Asian
Bor-Kucukatay et al. (2008) [14]	Turkey	20	45.1±3.07	45.8±2.9	14.7±0.7	14.4±0.8	NA	NA	Asian
Das et al. (2009) [15]	India	457	40.8±4.0	38.9±3.4	13.9±1.1	12.6±4.74	NA	NA	Indian
Moog (2009) [16]	Germany	60	42.6±3.0	42.9±3.6	14.6±1.0	14.6±1.6	4.7±0.4	4.7±0.4	Caucasian
Tendulkar et al. (i)* (2009) [17]	India	121	41.6±3.5	40.6±3.3	13.7±1.0	13.4±1.1	NA	NA	Indian
Tendulkar et al. (ii)* (2009) [17]	India	50	41.4±2.8	39.2±3.0	13.6±1.0	12.9±1.1	NA	NA	Indian
Tendulkar et al. (iii)* (2009) [17]	India	66	43±2.6	41.7±3.1	14.1±0.9	13.7±1.1	NA	NA	Indian
Mahmood et al. (2011) [18]	Malaysia	76	44.6±2.5	44.1±2.6	14.9±0.9	14.7±1.0	NA	NA	Asian
Heuft et al. # (2012) [19]	Germany-Austria	185	43.4±3.3	43.3±7.0	14.5±1.1	14.2±2.3	NA	NA	Caucasian
Heuft et al. ## (2012) [19]	Germany-Austria	226	43.2±3.3	43.3±4.0	14.4±1.1	14.4±1.3	NA	NA	Caucasian
Macher et al. ($) (2012) [20]	Austria	24	44.9±2.9	40.9±2.9	15.4±1.3	14.1±1.3	5.2±0.4	4.7±0.4	Caucasian
Macher et al. ($$) (2012) [20]	Austria	24	44.4±2.6	40.8±3.1	15.2±1.1	13.9±1.3	5.1±0.4	4.7±0.45	Caucasian
Patidar et al. (2012) [21]	India	500	40.2±1.7	36.2±2.3	13.3±0.6	12.1±0.8	NA	NA	Indian
Nomani et al. (2013) [22]	India	60	43.9±2.6	41.2±2.7	13.4±0.8	12.4±0.8	NA	NA	Indian
Sachdeva et al. (2014) [23]	India	171	42.7±3.4	43.4±3.6	15.3±1.2	15.6±1.3	NA	NA	Indian
Suresh et al. (2014) [24]	India	90	43.29±6.62	41.64±4.96	14.8±1.09	14.5±1.4	5.8±0.41	4.96±0.67	Indian
Gite et al. (2015) [25]	India	100	40.9±2.4	39.8±2.7	13.7±1.2	12.9±1.2	5.1±0.5	4.9±0.6	Indian
Farahat et al. (2016) [26]	Egypt	72	44.1±3.4	42.9±3.9	14.7±1.3	14.3±1.3	5±0.5	4.9±0.6	Caucasian
Landzo et al. (@) (2017) [27]	Croatia	60	43.4±2.7	43.7±2.6	15.4±1.0	14.6±1.1	NA	NA	Caucasian
Landzo et al. (@@) (2017) [27]	Croatia	60	44.4±2.8	41.3±2.8	14.3±1.0	14.3±1.0	NA	NA	Caucasian
Khurshid et al. (2020) [28]	India	100	45±3.56	42.3±3.93	15.3±1.33	14.4±1.47	NA	NA	Indian
Neha et al. (2022) [29]	India	150	42.94±3.43	43.28±3.55	14.59±1.17	14.74±1.2	5.06±0.44	5.13±0.43	Indian

**Figure 5:**
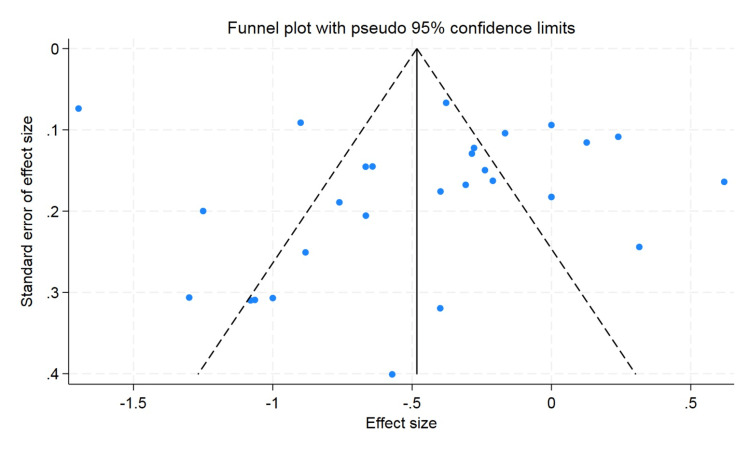
Funnel plot for hemoglobin.

**Figure 6:**
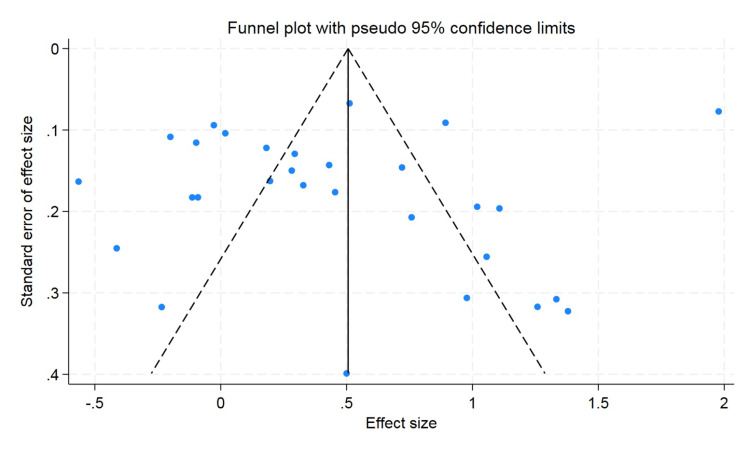
Funnel plot for hematocrit.

**Figure 7:**
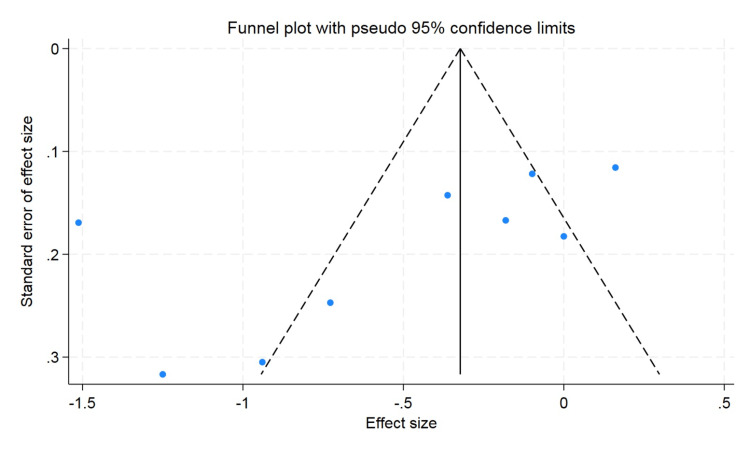
Funnel plot for erythrocyte.

A funnel plot was used to rule out publication bias, which did not observe the statistically significant publication bias. The p value for hemoglobin was 0.57 and 0.47 for hematocrit, and the p value for erythrocyte count was found to be 0.09, which showed that there was no publication bias (Figures 5-7).

Meta-Regression Analysis

**Figure 8:**
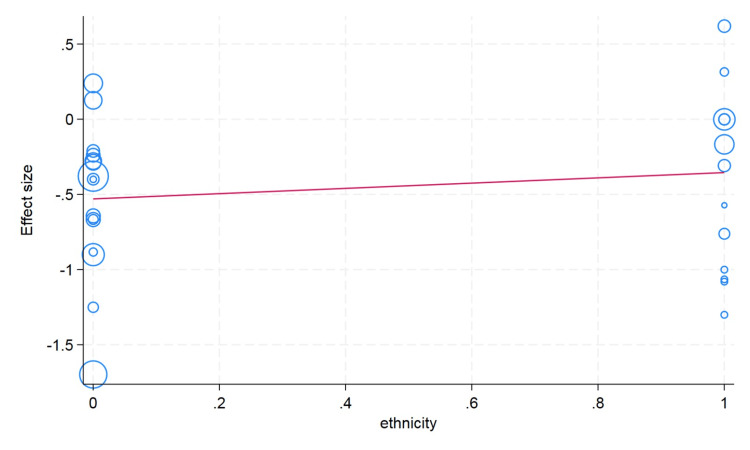
Meta-regression analysis graph showing the effect of ethnicity on hemoglobin. In the horizontal axis, 0 in ethnicity denotes Asian and 1 as Caucasian.

**Figure 9:**
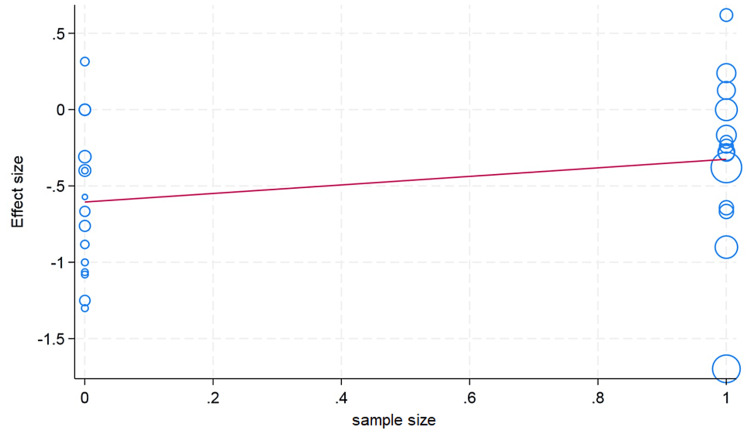
Meta-regression analysis graph showing the effect of the sample size on hemoglobin. In the horizontal axis, 0 is a sample size less than 75, whereas 1 is more than 75.

**Figure 10:**
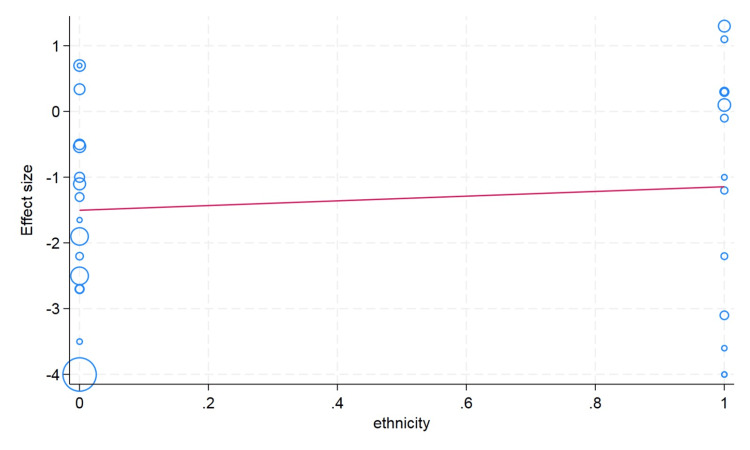
Meta-regression analysis graph showing the effect of ethnicity on hematocrit. In the horizontal axis, 0 in ethnicity denotes Asian and 1 as Caucasian.

**Figure 11:**
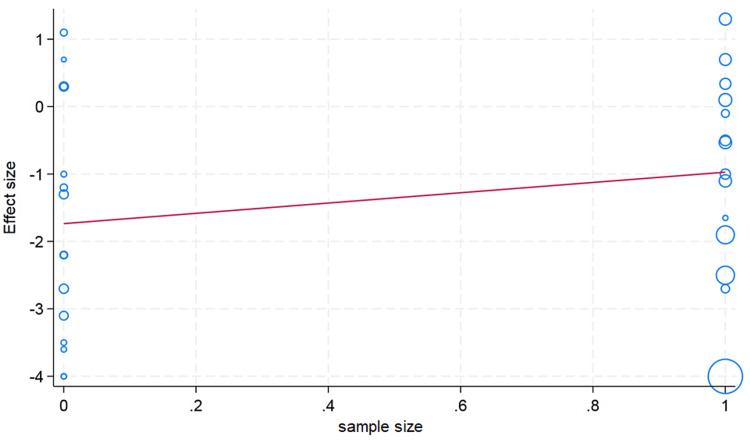
Meta-regression analysis graph showing the effect of sample size on hematocrit. In the horizontal axis, 0 is a sample size less than 75, whereas 1 is more than 75.

**Figure 12:**
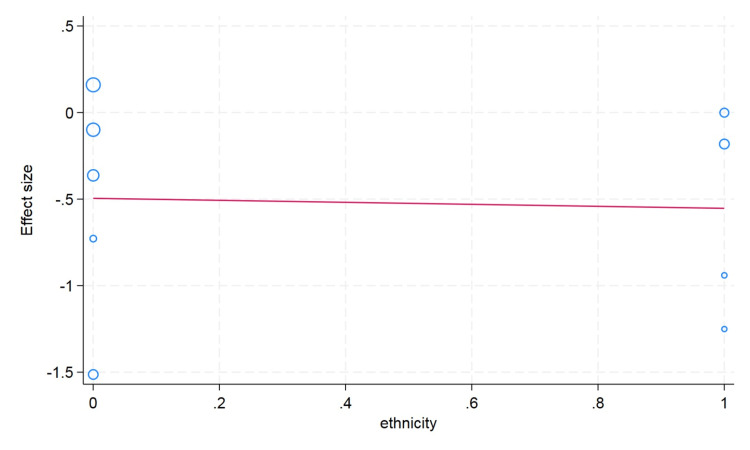
Meta-regression analysis graph showing the effect of ethnicity on erythrocyte count. In the horizontal axis, 0 in ethnicity denotes Asian and 1 as Caucasian.

**Figure 13:**
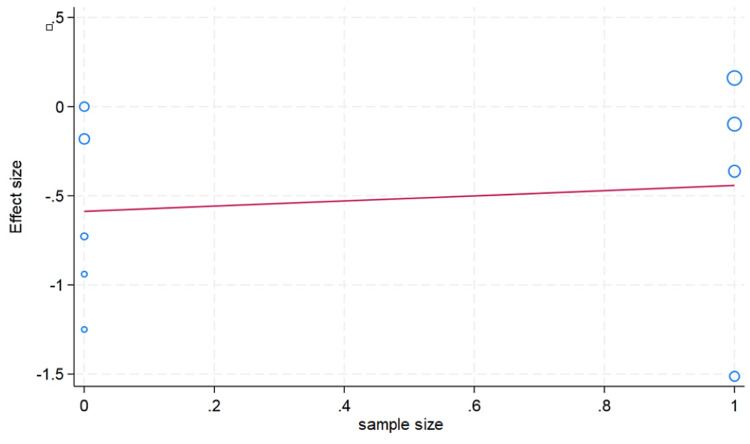
Meta-regression analysis graph showing the effect of sample size on erythrocyte counts. In the horizontal axis, 0 is a sample size less than 75, whereas 1 is more than 75.

We conducted a meta-regression analysis to check the moderator or confounding effects of clinically and demographically important variables on the effect size. We did not observe the moderator effect caused by ethnicity difference (Asian/Caucasian) on the effect size of hematocrit change (p value = 0.59). We observed that an increase in age in the included studies is associated with a decrease in change of the hematocrit value; however, this difference was not statistically significant (p value = 0.26). The sample size cutoff (more than 75) was not associated with the hematocrit value in the meta-regression analysis (p value = 0.24). In the erythrocyte, our meta-regression analysis did not note the significant influence of the sample size (p value = 0.74) and ethnicity (p value = 0.89) on the effect size of the change in erythrocyte value. However, with a very limited number of studies reported, the erythrocyte value and mean age showed that higher mean age in the individual studies had more decrease in the erythrocyte value (p value = 0.20). For the change in the hemoglobin value, the higher mean age in the individual studies showed less change in the hemoglobin value, but this difference was not statistically significant (p value = 0.13). We did not observe the moderator effects of the ethnicity (p value = 0.41) and sample size (p value = 0.17) cutoff on the pooled effect size of the change in hemoglobin value after plateletpheresis (Figures 8-13).

Discussion

Plateletpheresis has become a boon in the field of transfusion medicine. Platelet transfusions are needed either prophylactically or therapeutically. With the recent advance, double and even triple doses of platelets can be collected in a shrinking donor population. However, plateletpheresis has a great impact on donor hematological parameters, and with its increasing use, donor safety is an area of concern [3].

In our meta-analysis, 24 studies were included that evaluated the effect of plateletpheresis on the hemoglobin, hematocrit, and erythrocyte count of the donor. The number of donors that were evaluated was 3,374, 3,374, and 690, respectively. Meta-analyses provide precise estimates due to increasing power to detect effect size. A key step in meta-analyses involves evaluating and quantifying heterogeneity using statistical methods. It provides a comprehensive synthesis of existing evidence.

There was significant heterogeneity noticed between the studies for which potential reason could be differences in the donor population characteristics and a huge variation in the reported year of publication. The other source of variation could be older equipment use, which may be associated with more errors. In our study, it was found that the majority of publications were from India as there is a requirement for recurrent blood transfusion in India due to hemoglobinopathies, which are very prevalent in the subcontinent [30]. Most of the publications were published after the year 2000, showing the increasing interest in the importance of apheresis as well as concern for the role of the safety of the donors. We presented the forest plot showing that, in the majority of the studies, platelet donation by apheresis caused hemoglobin, hematocrit, and erythrocyte values to decrease. The most important cause that led to a decrease in the hematological parameters in this meta-analysis was that the majority of the machines used a saline infusion technique during plateletpheresis, which led to hemodilution that caused the decrease in the value of hematological parameters such as hemoglobin, hematocrit, and erythrocyte. Another reason was that, after the procedure of plateletpheresis, a small volume of blood was left behind in the apheresis kit. Thirdly, during plateletpheresis, extracorporeal hemolysis occurs. It is due to the exposure of erythrocytes to stress or change in osmotic pressure. Additionally, there is reduced donor platelet reserve or altered megakaryopoietic homeostasis in repeated plateletpheresis donors.

Das et al. mentioned the differential effect of the cell separators used on the post-donation cell counts. They found more platelet and RBC loss in the first-generation apheresis devices as compared to the more recent ones [15]. Beyan et al. also found a significant difference in the decrease in hemoglobin and hematocrit in the different cell separators they compared [9]. In the study by Lazarus et al. and Farahat et al., where there were donors with repeated donation, there was a reduced platelet reserve and altered megakaryopoietic homeostasis [6,26].

In the studies done by Love et al., Sachdeva et al., and Neha et al., there was an increase in the hematological parameters after plateletpheresis, which could be due to the machine that was used, in which less volume of blood was left in the apheresis kit and the sample collected where within 30 minutes after plateletpheresis, which caused an increase in the hematological parameters because physiological compensation could not take place within 30 minutes [8,23,29].

In meta-regression analysis, we observed that a higher mean age in individual studies had a greater decrease in the erythrocyte value. This could be due to a limited number of studies that reported data on erythrocyte value and mean age. Additionally, there is a decline in bone marrow cellularity with increasing age.

The various areas of concern that need to be addressed are citrate-related complications, such as shivering, nausea, chills, abdominal pain, severe hypocalcemia that may progress to frank tetany including life-threatening laryngospasm that is mainly seen in older and low body weight donors, and the risk of a transient pro-thrombotic state due to the extracorporeal contact of blood, with plastic surfaces raising the concern for donor-related safety [20,26]. Nevertheless, the study can help in establishing the reference ranges for a procedure that can improve the production of platelets and the safety of platelet donors.

This study helps in determining the factors that can put donors at high risk, which can adversely affect voluntary donor recruitment and retention strategies. Hereby, there is a need to create awareness in the public regarding the constant need for blood and blood products. In developing countries, such as India, first-time donors are a major population of plateletpheresis donors, and they should be retained as future voluntary repeat donors.

Limitations

The study had several limitations to generalize the findings. Physiological data of the donors were not available to study to detect the effects of other variables in the study outcome. The number of previous donations of donors was unknown to see its moderator effect in the effect size. Effects of the procedure on serum ferritin level were not available in the included to see its confounding effects on the size of the effect. The unavailability of other anthropometric data did not allow to conduct of moderator effects for these variables. Different analyzers give different values of platelets because of their different principle, and hence analyzer values of platelets were not considered. Heterogeneity across studies is seen because of differences in study protocols that cause potential bias.

## Conclusions

Plateletpheresis shows the fallout of the hemoglobin, hematocrit, and erythrocyte count due to blood loss in the kits employed in the procedure and cell lysis because of exposure of erythrocytes to stress or change in osmotic pressure. By improving the automated machines for plateletpheresis, the quality of products can be increased, and the duration of procedures for the betterment of donor health can be decreased. There needs to be the development of strict criteria for donation for better safety of donors and standardized reporting of important hematological parameters (hemoglobin, hematocrit, RBCs, and platelets). In the future, large, well-designed studies should be conducted.
